# West Nile virus outbreak among horses in New York State, 1999 and 2000.

**DOI:** 10.3201/eid0704.010427

**Published:** 2001

**Authors:** S C Trock, B J Meade, A L Glaser, E N Ostlund, R S Lanciotti, B C Cropp, V Kulasekera, L D Kramer, N Komar

**Affiliations:** New York State Department of Agriculture and Market, 1 Winners Circle, Albany, NY 12235, USA. Susan.Trock@agmkt.state.ny.us

## Abstract

West Nile (WN) virus was identified in the Western Hemisphere in 1999. Along with human encephalitis cases, 20 equine cases of WN virus were detected in 1999 and 23 equine cases in 2000 in New York. During both years, the equine cases occurred after human cases in New York had been identified.


### West Nile Virus Outbreak Among Horses in New York State, 1999 and 2000

**Susan C. Trock,* Barry J. Meade,† Amy L. Glaser,* Eileen N. Ostlund,‡ Robert S. Lanciotti,§ Bruce C. Cropp,§ Varuni Kulasekera,¶ Laura D. Kramer,** and Nicholas Komar§**

*Cornell University, Veterinary Diagnostic Laboratory, Ithaca, New York, USA; †U.S. Department of Agriculture (USDA), Veterinary Services, Frankfort, Kentucky, USA; ‡USDA, National Veterinary Services Laboratories, Ames, Iowa, USA; §Centers for Disease Control and Prevention, Fort Collins, Colorado, USA; ¶New York City Department of Health, New York City, New York, USA; **Arbovirus Research Laboratory, Wadsworth Center, Albany, New York, USA

West Nile (WN) virus was identified in the Western Hemisphere in 1999. Along with human encephalitis cases, 20 equine cases of WN virus were detected in 1999 and 23 equine cases in 2000 in New York. During both years, the equine cases occurred after human cases in New York had been identified.

An outbreak of arboviral encephalitis attributable to West Nile (WN) virus was first recognized in the United States in 1999, when dead crows were reported in and near New York City, and a zoological park noted that some of their exhibition birds had died. These events coincided with initial public health reports of human encephalitis cases diagnosed as St. Louis encephalitis virus in New York City (1). The successful isolation of virus from dead birds allowed the subsequent identification of WN virus as the etiologic agent of both human and animal disease (2).

WN virus is primarily transmitted between mosquitoes and birds, but transmission to mammals can occur when infection occurs in mosquito species that feed on birds and mammals. Encephalitis has been confirmed only in humans and horses (1,3-10). During 1999, 20 equine cases of WN virus encephalitis were confirmed in the United States, all in New York. In 2000, 23 equine cases were identified in New York, with more cases identified in New Jersey, Delaware, Rhode Island, Massachusetts, Connecticut, and Pennsylvania. We summarize these findings.

### The Study

### 1999 Investigations

During fall and early winter 1999, veterinarians with the U.S. Department of Agriculture and the New York Department of Agriculture and Markets (NYSDAM) investigated reports of an unusual cluster of neurologic illness in horses on Long Island. Investigations were initiated by reports from practicing veterinarians. Other veterinarians were contacted to determine whether other similar cases were occurring in the area. Tissue and blood samples were collected and submitted to the National Veterinary Services Laboratories (NVSL) for testing and forwarded to the Centers for Disease Control and Prevention (CDC) for confirmation, as necessary.

Address for correspondence: Susan C. Trock, New York State Department of Agriculture and Market, 1 Winners Circle, Albany, NY 12235; fax: 518-485-7773; e-mail: Susan.Trock@agmkt.state.ny.us

In 1999, a case was defined as an equine with neurologic signs and either a positive plaque reduction neutralization test (PRNT) titer to WN virus or isolation of virus confirmed by primer sequence. Testing was conducted at either CDC or the NVSL (11). Testing for immunoglobulin M (IgM) antibody was not done.

Twenty cases of equine neurologic illness (1 pony and 19 horses, all from Long Island) were laboratory confirmed as WN virus infections. Five additional horses with clinical onset between August 28 and September 24, before the cluster was reported, were considered probable cases. The illness, characterized by acute onset of rear limb ataxia, included muscle tremors, knuckling over at the fetlocks, and in some instances inability to rise. The first horse became ill on August 26; the onset of the last case was October 23 (Figure 1).

Four of the 20 animals died or were euthanized. All survived for 3 or 4 days before euthanasia. Necropsy samples from three of these horses yielded WN virus from brain or spinal cord tissue. The fourth horse had a WN virus titer (>1:320) from a sample collected 3 days after clinical onset. The four dead horses ranged in age from 4 to 21 years old. Sixteen of the horses recovered fully and had neutralizing antibody titers to WN virus from ≥1:100 (NVSL) to ≥1:1,280

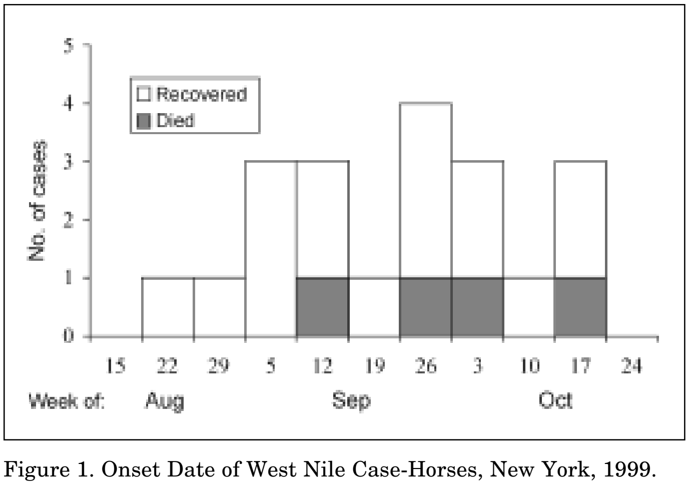

Figure 1. Onset Date of West Nile Case-Horses, New York, 1999.

(CDC). The 20 animals ranged in age from 2 to 28 years old. There were 13 mares, 3 geldings, and 4 stallions.

The 20 cases and 5 probable cases lived on 18 different premises in Nassau or Suffolk counties. Stable mates were identified on 15 of these premises. Samples were collected from 69 asymptomatic stable mates. Of these, 20 (29%) had titers to WN virus ranging from 1:160 to $\geq$1:1,280. The age of these horses (1 to 37 years old) did not differ significantly from case-horse ages. There were 27 mares, 32 geldings, and 8 stallions.

### 2000 Investigations

On May 16, 2000, an informational letter, signed by the NYSDAM and the New York State Department of Public Health (NYSDOH), was sent to approximately 1,600 New York veterinarians. The letter summarized the 1999 findings and requested case reporting of suspected equine cases to NYSDAM and suspected cases in companion animals to NYSDOH.

In 2000, 23 WN virus encephalitis cases in horses were confirmed in New York State. Diagnostic samples were submitted to the New York State Animal Disease Diagnostic Laboratory (NYSADDL) in Ithaca. All positive diagnoses were based on IgM antibody and positive WN virus neutralization, a demonstrated fourfold rise in virus neutralization titer from paired serum samples, or detection of viral sequence by reverse transcription-polymerase chain reaction (RT-PCR) performed at the NVSL. One horse was diagnosed by real-time RT-PCR testing at the Arbovirus Laboratory of the Wadsworth Center, NYSDOH. Although no infectious virus was grown in Vero cell culture, the brain sample was also positive by two primer-probe sets (1160 and 3111).

The first equine case had clinical onset on August 18 and lived on Staten Island. The last had onset on November 1 (Figure 2); onset date could not be determined for one horse.

The index horse had positive titers (IgM 1:1,000 and PRNT $\geq$1:100) for WN virus; six other horses had the same titers. Nine cases had IgM titers of 1:10 and PRNT titers of $\geq$1:1,000. Six horses had various combinations of IgM (1:10 to 1:100) and PRNT ($\geq$1:100) titers. One horse, with negative serology, was diagnosed by RT-PCR by the NYSDOH Arbovirus Laboratory. This brain sample was also positive by two primer-probe sets.

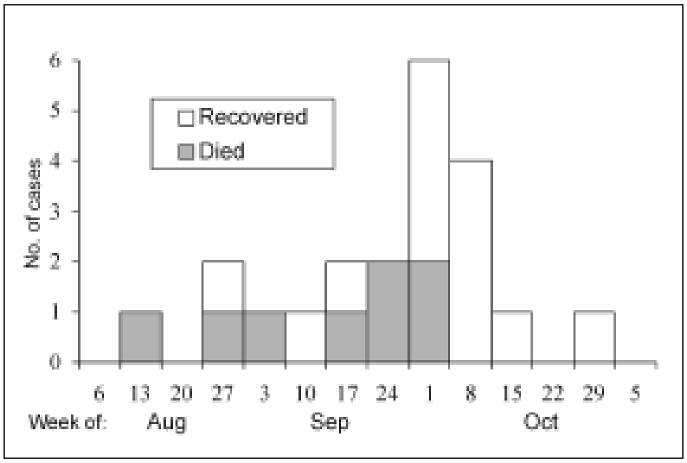

Figure 2. Onset Date of West Nile Case-Horses—New York, 2000.

Horses had ataxia (95.7%), primarily rear limb (90.5%,) and muscle fasciculations or trembling (55%). Many had acute onset (90.5%). Other case-horses were down; some were able to rise with assistance, while others could not stand. Only 32% of the horses had elevated temperatures (Table). Eight horses died or were euthanized; seven died within 3 to 4 days of clinical onset. The average age of horses with fatal cases was 14.4 years, similar to the age of surviving horses. No significant gender differences or breed predispositions for disease were detected. Cases occurred in Suffolk, Orange, Nassau, Bronx, and Richmond counties.

Attempts were made to contact veterinarians who submitted samples to the NYSADDL for WN virus testing on equine sera. When reached, veterinarians were interviewed to determine if the submission was for diagnostic purposes and asked to provide the clinical picture of the ill horse. Only horses with primarily a neurologic component were included in the study (e.g., lameness was excluded). Clinical findings of the 23 case-horses were compared with those of 19 non-case horses with similar clinical signs but with no laboratory evidence of WN virus infection (Table). No statistically significant difference was found. The average age of the non-case horses was 14.4 years (range 2-28). Results indicated that WN virus cannot be diagnosed on the basis of clinical signs alone.

A serosurvey of horses on Staten Island was conducted during September 2000. Ninety-one clinically normal horses from seven stables located within 3 miles of the index case-horse were sampled. Seven seropositive horses were identified at three stables, including the stable mate of the index horse. At one stable, five of six horses sampled had titers to WN virus; one of the five had a positive IgM-capture enzyme-linked immunosorbent assay (MAC-ELISA)(1:100) and PRNT positive at 1:10. Sera from the other four horses were negative by MAC-ELISA but positive at 1:100 with the PRNT at NVSL. Sera from the two other positive horses at the other two stables were negative by MAC-ELISA but positive at 1:10 by PRNT.

Mosquito surveillance conducted by the New York City Department of Health (NYCDOH) within a 2-mile radius of

Table. Horses with neurologic illness—New York, 2000

| Sign | Number of case-horses[a] | Number of non-case horses[b] |
|---|---|---|
| Ataxia | 22/23 (95.7%) | 16/19 (84.2%) |
| Rear | 19/21 (90.5%) | 15/19 (78.9%) |
| All four limbs | 15/20 (75%) | 9/19 (47.4%) |
| Acute onset | 19/21 (90.5%) | 12/18 (66.7%) |
| Fever (>101°F) | 7/22 (31.8%) | 8/15 (60%) |
| Mean | 102.4°F | 104°F |
| Range | 101.4-103 | 102-106 |
| Muscle fasciculation | 11/20 (55%) | 5/19 (26.3%) |
| Almost fall[c] | 8/17 (47.1%) | 6/18 (33.3%) |
| Down | 9/22 (40.9%) | 4/17 (23.5%) |
| Rise with assist | 6/21 (28.6%) | 5/18 (27.8%) |
| Died | 8/23 (34.8%) | 8/19 (42.1%) |
| Dull, lethargic | 5/19 (26.3%) | 10/19 (52.6%) |
| Hypermetric | 4/16 (25%) | 4/19 (21.1%) |
| Agitated | 3/19 (15.8%) | 2/18 (11.1%) |

[a]West Nile (WN) virus confirmed by laboratory testing. Denominator varies depending upon completeness of information provided during interview.
[b]WN virus negative by laboratory testing. Denominator varies depending upon completeness of information provided during interview.
[c]Private practitioners reporting that circling the horse would cause it to fall.

the three positive premises from July to November resulted in 44 WN virus-positive pools. These included *Culex pipiens*, which feed only on birds; *Cx. salinarius*, which feed on both birds and mammals; and *Aedes vexans*, *Ae. triseriatus*, and *Psorophora ferox*, which feed mainly on mammals. In July, *Cx. salinarius* was the only positive mosquito pool in this radius. One week after the index horse became ill, the NYCDOH began trapping mosquitoes at its stable; WN virus was identified in a pool of *Ae. vexans* 10 days after the horse became ill. This was the first identification of this mosquito as positive for WN virus in New York.

### Conclusions

In 2000 (unlike in 1999, when resources for laboratory diagnosis of flavivirus infections were limited), MAC-ELISA and real-time RT-PCR were available to detect WN-specific IgM antibodies and WN virus RNA, respectively.

WN infection in horses may cause acute, fatal neurologic disease, but clinical disease often does not occur. Moderate to severe ataxia, weakness, and rear limb incoordination were the most consistent signs; fever was not. Treatment was primarily directed toward relieving clinical signs. In some instances in which the horses were only mildly affected, no treatment was given, and clinical signs resolved in 2 to 7 days. Horses that survived eventually recovered fully.

The epidemic curves of the 1999 and 2000 equine outbreaks are similar, with equine cases occurring after human and wild bird cases. Horses are unlikely to be the first warning that virus is circulating in an area. In each instance where equine cases occurred, virus activity in wild birds, mosquitoes, or both had already been identified.

There were two case-horses on Staten Island, where 10 human cases occurred (12). No other NY counties with equine cases reported human cases in 1999 or 2000.

Unlike with human cases, veterinarians cannot rely on fever to aid in diagnosing WN virus in horses. In addition, clinical presentation of WN virus in horses is nonspecific. For Staten Island in 2000, an area of intense WN virus activity, equine cases did not serve as an early warning to public health officials that virus was circulating. In other NY counties, equine cases did not precede WN findings in mosquitoes and wild birds, nor did they predict human cases.

### Acknowledgments

We thank J. Velez for technical work on specimens; D.J. Johnson, D.D. Pedersen, K. Moser, and K. Lake for test development, virus isolation, and serology testing; K.A. Bernard, E.B. Kauffman, G.D. Ebel, J. Huntley, S.A. Jones, A.P. Dupuis II, J.G. Maffei, K.A. Ngo, J. Pomakoy, D. Young, and D.C. Nicholas for technical work; and J.R. Miller and B. Cherry for assistance with data gathering.

Dr. Trock is the epidemiologist at Cornell University assigned to the New York State Department of Agriculture and Markets in Albany. After training with the Centers for Disease Control and Prevention's Epidemic Intelligence Service, she has conducted field investigations of various zoonotic and animal infectious disease agents.

### References

1. Centers for Disease Control and Prevention. Outbreak of West Nile-like viral encephalitis--New York, 1999. MMWR Morb Mortal Wkly Rep 1999;48:845-9.
2. Centers for Disease Control and Prevention. Update: West Nile-like viral encephalitis--New York, 1999. MMWR Morb Mortal Wkly Rep 1999;48:890-2.
3. Cantile C, Di Guardo G, Eleni C, Arispici M. Clinical and neuropathological features of West Nile virus equine encephalomyelitis in Italy. Equine Vet J 2000;32:31.
4. Ceausu E, Erscoiu S, Calistru P, Ispas D, Dorobat O, Homos M, et al. Clinical manifestations in the West Nile virus outbreak. Rom J Virol 1997;48:3-11.
5. Cernescu C, Ruta SM, Tardei G, Grancea C, Moldoveanu L, Spulbar E, et al. A high number of severe neurologic clinical forms during an epidemic of West Nile virus infection. Rom J Virol 1997;48:13-25.
6. Lvov DK, Butenko AM, Gromashevsky VL, Larichev VP, Gaidamovich SY, Vyshemirsky OI, et al. Isolation of two strains of West Nile virus during an outbreak in southern Russia, 1999. Emerg Infect Dis 2000;6:373-6.
7. Nur YA, Groen J, Heuvelmans H, Tuynman W, Copra C, Osterhaus AD. An outbreak of West Nile fever among migrants in Kisangani, Democratic Republic of Congo. Am J Trop Med Hyg 1999;61:885-8.
8. Pantheir R, Hannoun C, Oudar J, Beytout D, Corniou B, Joubert L, et al. Isolation of West Nile virus in a Camarge horse with encephalomyelitis. Comptes Rendus Hebdomadaires Des Seances de L'Academie des Sciences D: Sciences Naturelle (Paris) 1966;262:1308-10.
9. Panthier R. Epidemiology of the West Nile virus: study of an outbreak in Camargue. I. Introduction. Ann Inst Pasteur (Paris) 1968;114:518-20.
10. Tsai TF, Popovici F, Cernescu C, Campbell GL, Nedelcu NI. West Nile encephalitis epidemic in southeastern Romania. Lancet 1998;352:76771.
11. Beaty BJ, Calisher CH, Shope RE. Arbovirus. In: Schmidt NH, Emmons RW, editors. Diagnostic procedures for viral, rickettsial and chlamydial infections. 6th ed. Washington: American Public Health Association 1989;797-856.
12. Kulasekera V, Kramer L, Nasci R, Mostashari F, Cherry B, Trock SC, et al. West Nile virus infection in mosquitoes, birds, horses, and humans, Staten Island, New York, 2000. Emerg Infect Dis 2001;7:722-5.
